# Supplementary material for: Pyruvate Dehydrogenase Kinase 1 inhibition mediated oxidative phosphorylation enhancement in cartilage promotes osteoarthritis progression
Source: BMC Musculoskelet Disord. 2023 Jul 20;24:597. doi: 10.1186/s12891-023-06585-6 (PMC10357736; doi:10.1186/s12891-023-06585-6)
Supplement: Supplementary file 1 — Additional file 1. [file 12891_2023_6585_MOESM1_ESM.zip › BP.Gsea.1653623667859/GOBP_PROTEIN_INSERTION_INTO_ER_MEMBRANE.html]

Details for gene set GOBP\_PROTEIN\_INSERTION\_INTO\_ER\_MEMBRANE[GSEA]

|  || Dataset | 55457\_collapsed\_to\_symbols.cls.cls #OA\_versus\_normal.cls.cls #OA\_versus\_normal\_repos |
| Phenotype | cls.cls#OA\_versus\_normal\_repos |
| Upregulated in class | OA |
| GeneSet | GOBP\_PROTEIN\_INSERTION\_INTO\_ER\_MEMBRANE |
| Enrichment Score (ES) | 0.60148984 |
| Normalized Enrichment Score (NES) | 1.8878789 |
| Nominal p-value | 0.0 |
| FDR q-value | 0.04822224 |
| FWER p-Value | 0.796 |
Table: GSEA Results Summary

  

Fig 1: Enrichment plot: GOBP\_PROTEIN\_INSERTION\_INTO\_ER\_MEMBRANE      
 Profile of the Running ES Score & Positions of GeneSet Members on the Rank Ordered List

  

| SYMBOL | TITLE | RANK IN GENE LIST | RANK METRIC SCORE | RUNNING ES | CORE ENRICHMENT || 1 | GET1 | guided entry of tail-anchored proteins factor 1 [Source:HGNC Symbol;Acc:HGNC:12790] | 159 | 0.852 | 0.0862 | Yes |
| 2 | WDR83OS | WD repeat domain 83 opposite strand [Source:HGNC Symbol;Acc:HGNC:30203] | 239 | 0.791 | 0.1715 | Yes |
| 3 | EMC9 | ER membrane protein complex subunit 9 [Source:HGNC Symbol;Acc:HGNC:20273] | 276 | 0.768 | 0.2576 | Yes |
| 4 | UBL4A | ubiquitin like 4A [Source:HGNC Symbol;Acc:HGNC:12505] | 464 | 0.675 | 0.3211 | Yes |
| 5 | EMC7 | ER membrane protein complex subunit 7 [Source:HGNC Symbol;Acc:HGNC:24301] | 468 | 0.673 | 0.3987 | Yes |
| 6 | TRAM1 | translocation associated membrane protein 1 [Source:HGNC Symbol;Acc:HGNC:20568] | 569 | 0.633 | 0.4641 | Yes |
| 7 | EMC1 | ER membrane protein complex subunit 1 [Source:HGNC Symbol;Acc:HGNC:28957] | 1067 | 0.502 | 0.4835 | Yes |
| 8 | EMC2 | ER membrane protein complex subunit 2 [Source:HGNC Symbol;Acc:HGNC:28963] | 1096 | 0.497 | 0.5388 | Yes |
| 9 | GET3 | "guided entry of tail-anchored proteins factor 3, ATPase [Source:HGNC Symbol;Acc:HGNC:752]" | 1161 | 0.485 | 0.5899 | Yes |
| 10 | EMC3 | ER membrane protein complex subunit 3 [Source:HGNC Symbol;Acc:HGNC:23999] | 1624 | 0.410 | 0.6015 | Yes |
| 11 | EMC6 | ER membrane protein complex subunit 6 [Source:HGNC Symbol;Acc:HGNC:28430] | 2839 | 0.266 | 0.5380 | No |
| 12 | CAMLG | calcium modulating ligand [Source:HGNC Symbol;Acc:HGNC:1471] | 3668 | 0.188 | 0.4956 | No |
| 13 | TRAM2 | translocation associated membrane protein 2 [Source:HGNC Symbol;Acc:HGNC:16855] | 4913 | 0.079 | 0.4081 | No |
| 14 | BAG6 | BAG cochaperone 6 [Source:HGNC Symbol;Acc:HGNC:13919] | 5023 | 0.071 | 0.4079 | No |
| 15 | SEC61A1 | SEC61 translocon subunit alpha 1 [Source:HGNC Symbol;Acc:HGNC:18276] | 5378 | 0.041 | 0.3852 | No |
| 16 | EMC8 | ER membrane protein complex subunit 8 [Source:HGNC Symbol;Acc:HGNC:7864] | 6104 | -0.013 | 0.3304 | No |
| 17 | SGTA | small glutamine rich tetratricopeptide repeat co-chaperone alpha [Source:HGNC Symbol;Acc:HGNC:10819] | 11185 | -0.455 | -0.0113 | No |
| 18 | EMC10 | ER membrane protein complex subunit 10 [Source:HGNC Symbol;Acc:HGNC:27609] | 11497 | -0.501 | 0.0225 | No |
| 19 | CCDC47 | coiled-coil domain containing 47 [Source:HGNC Symbol;Acc:HGNC:24856] | 12495 | -0.746 | 0.0314 | No |
Table: GSEA details [plain text format]

  

Fig 2: GOBP\_PROTEIN\_INSERTION\_INTO\_ER\_MEMBRANE      
 Blue-Pink O' Gram in the Space of the Analyzed GeneSet

  

Fig 3: GOBP\_PROTEIN\_INSERTION\_INTO\_ER\_MEMBRANE: Random ES distribution      
 Gene set null distribution of ES for **GOBP\_PROTEIN\_INSERTION\_INTO\_ER\_MEMBRANE**
